# Supplementary material for: αV-Integrin-Dependent Inhibition of Glioblastoma Cell Migration, Invasion and Vasculogenic Mimicry by the uPAcyclin Decapeptide
Source: Cancers (Basel). 2023 Sep 28;15(19):4775. doi: 10.3390/cancers15194775 (PMC10571957; doi:10.3390/cancers15194775)
Supplement: Supplementary file 1 [file cancers-15-04775-s001.zip › Supplementary information-Peptide Synthesis.pdf]

Supplementary information- Experimental procedure

Synthesis of the decapeptides, acetylation, and FITC-conjugation

The peptide sequences were assembled by the Fmoc-based US-SPPS method. The Fmoc Rink amide resin (0.1 mmol; 0.72 mmol/g as loading, 100-200 mesh as particle size) was swollen in DMF for 20 min. Then, a 20% piperidine in DMF solution was added to remove the Fmoc group, thus the tube reactor was placed in an ultrasonic bath with the reaction mixture not exceeding the water level (0.5 + 1 min). After each step, filtering and washings of the resin were executed (3 × 2 mL of DMF). Couplings were performed by treatment with a solution of the Fmoc-amino acid (2 equiv), COMU (2 equiv), Oxyma (2 equiv), and DIEA (4 equiv) in DMF, and exposing the resin to the ultrasonic irradiation for 5 min. *N*-The terminal ends of **uPAcyclin** and **S-uPAcyclin** were acetylated by treatment with a solution of Ac_2O (2 equiv) and DIPEA (4 equiv) in DMF, and shaking the reactor on an automated shaker for 30 min at rt; whereas, **FITC-uPAcyclin** was labeled with fluorescein on the *N*-terminal by the treatment with fluorescein isothiocyanate (FITC) prior introduction of a spacer. To monitor both Fmoc deprotection and coupling reactions, the colorimetric Kaiser or chloranil tests were used for the detection of solid-phase bound primary and secondary amines, respectively.

Upon elongation, the resin-bound peptides were treated for the selective removal of the allyl-based protecting groups of Glu and Lys residues. In particular, resins were washed with DCM (3 × 2 mL), suspended in a solution of $Pd(PPh_3)_4$ (0.15 equiv) and NDMBA (3 equiv) in dry DCM/DMF (3:2, *v/v*), and gently shaken for 1 h under Ar. The resin was filtered, washed with DMF (3 × 2 mL) and DCM (3 × 2 mL), and dried. Such procedure was repeated for a second time. Finally, an additional washing with 0.5% sodium *N,N*-diethyldithiocarbamate solution in DMF (30 min × 2) was made, and the complete removal of the allyl groups was ascertained by LC-MS analysis. The released amine and carboxylic acid were thus coupled using PyAOP (2 equiv), HOAt (2 equiv), and DIEA (4 equiv), dissolved in DMF/DCM (1:1, *v/v*). The reaction mixture was shaken for 16 h at rt, and the conversion to the cyclic product was monitored by LC-MS analysis. Finally, the peptidyl resin was dried in vacuo and cleaved by treating with a 95% TFA solution, recovered by precipitation with Et_2O, and then centrifuged (6000 rpm × 15 min). The supernatants were carefully removed, and the resulting amorphous solids were dried and dissolved in water/acetonitrile (9:1) to be analyzed by reverse-phase HPLC.

Purification of peptides was performed by RP-HPLC (Shimadzu Preparative Liquid Chromatograph LC-8A) equipped with a preparative column (Phenomenex Kinetex C18 column, 5 μm, 100 Å, 150 × 21.2 mm) using linear gradients of MeCN (0.1% TFA) in water (0.1% TFA), from 10 to 90% over 30 min, with a flow rate of 10 mL/min and UV detection at 220 nm. Final products were obtained by lyophilization of the appropriate fractions after removal of the MeCN by rotary evaporation. Analytical UHPLC (Shimadzu Nexera Liquid Chromatograph LC-30AD) analyses to assess the purity of peptides were

performed on a Phenomenex Kinetex reversed-phase column (C18, 5 μm, 100 Å, 150 × 4.6 mm) with a flow rate of 1 mL/min using a gradient of MeCN (0.1% TFA) in water (0.1% TFA), from 10 to 90% over 15 min, and UV detection at 220 nm. All compounds examined for biological activity were purified to >95%, and prior their use for biological assays the correct molecular ions were confirmed by mass spectrometry.
