# Supplementary figures and images for: αV-Integrin-Dependent Inhibition of Glioblastoma Cell Migration, Invasion and Vasculogenic Mimicry by the uPAcyclin Decapeptide
Source: Cancers (Basel). 2023 Sep 28;15(19):4775. doi: 10.3390/cancers15194775 (PMC10571957; doi:10.3390/cancers15194775)

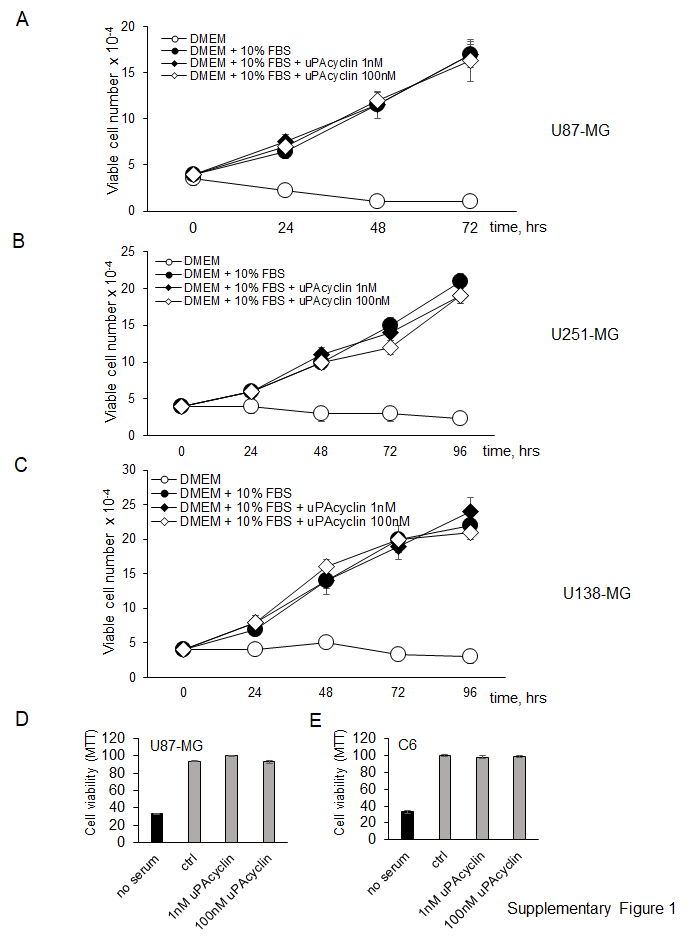

Supplement: Supplementary file 1 [file cancers-15-04775-s001.zip › Supplementary Figure S1_Franco et al _ copia.tif]

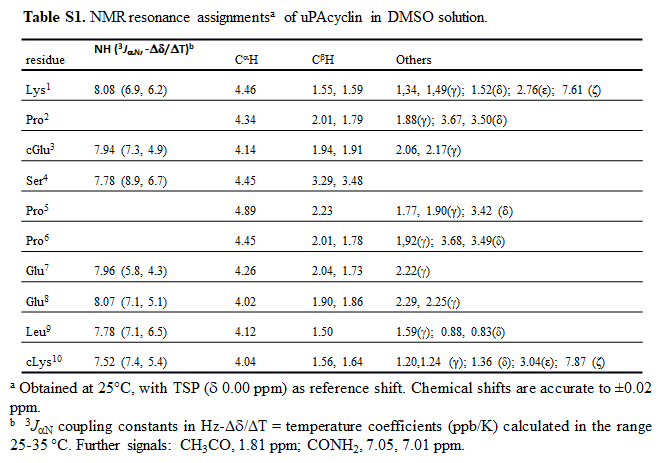

Supplement: Supplementary file 1 [file cancers-15-04775-s001.zip › Table S1_Franco et al _ copia.tif]

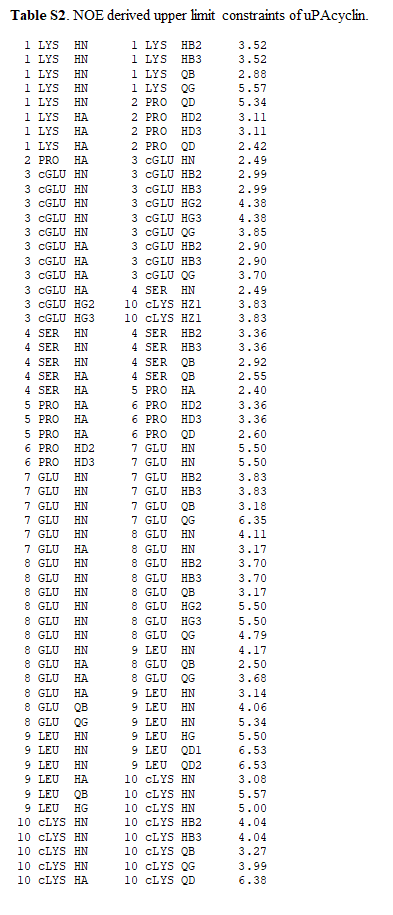

Supplement: Supplementary file 1 [file cancers-15-04775-s001.zip › Table S2_Franco et al _ copia.tif]
